# Transposition of Insertion Sequences was Triggered by Oxidative Stress in Radiation-Resistant Bacterium *Deinococcus geothermalis*

**DOI:** 10.3390/microorganisms7100446

**Published:** 2019-10-12

**Authors:** Chanjae Lee, Nakjun Choi, Min K. Bae, Kyungsil Choo, Sung-Jae Lee

**Affiliations:** Department of Biology, Kyung Hee University, Seoul 02447, Korea; qkektk456@naver.com (C.L.); dps02116@khu.ac.kr (N.C.); virginiakmb@gmail.com (M.K.B.); reginachoo@hanmail.net (K.C.)

**Keywords:** Insertion sequences, transposase, transposition, genomic plasticity, oxidative stress, *Deinococcus geothermalis*

## Abstract

During an oxidative stress-response assay on a putative Dps-like gene-disrupted Δ*dgeo*_0257 mutant strain of radiation-resistant bacterium *Deinococcus geothermalis*, a non-pigmented colony was observed among the normal reddish color colonies. This non-pigmented mutant cell subsequently displayed higher sensitivity to H_2_O_2_. While carotenoid has a role in protecting as scavenger of reactive oxygen species the reddish wild-type strain from radiation and oxidative stresses, it is hypothesized that the carotenoid biosynthesis pathway has been disrupted in the mutant *D. geothermalis* cell. Here, we show that, in the non-pigmented mutant cell of interest, phytoene desaturase (Dgeo_0524, *crt*I), a key enzyme in carotenoid biosynthesis, was interrupted by transposition of an IS*Dge*7 family member insertion sequence (IS) element. RNA-Seq analysis between wild-type and Δ*dgeo*_0257 mutant strains revealed that the expression level of IS*Dge*5 family transposases, but not IS*Dge*7 family members, were substantially up-regulated in the Δ*dgeo*_0257 mutant strain. We revealed that the non-pigmented strain resulted from the genomic integration of IS*Dge*7 family member IS elements, which were also highly up-regulated, particularly following oxidative stress. The transposition path for both transposases is a replicative mode. When exposed to oxidative stress in the absence of the putative DNA binding protein Dgeo_0257, a reddish *D. geothermalis* strain became non-pigmented. This transformation was facilitated by transposition of an IS*Dge*7 family IS element into a gene encoding a key enzyme of carotenoid biosynthesis. Further, we present evidence of additional active transposition by the IS*Dge*5 family IS elements, a gene that was up-regulated during the stationary phase regardless of the presence of oxidative stress.

## 1. Introduction

Genus *Deinococcus* is an aerobic Gram-positive bacterium capable of surviving in several extreme and/or harmful conditions (e.g., high levels of radiation, desiccation, oxidative stress, and starvation) [1,2,3]. The primary mechanisms mediating resistance to cellular stress are related to the ability of *Deinococcus* strains to protect damaged DNA and their proteome and other factors as follows: (1) structural organization of cell wall, (2) genome packaging, (3) active removal of toxic compounds, and (4) regulation of gene expression [4,5]. There are many known proteins for the protection of double strand DNA breakage and the DNA repair (eg, RecA, Ddr, and Ppr) [1,6,7]. Despite many studies using a myriad of molecular approaches to detect genus-specific DNA Damage Repair (DDR) machinery in *Deinococcus*, the protection and repair mechanisms of this genus remain largely unclear.

One DNA-protection protein involved in stress responses is the DNA-binding protein from starved cells (Dps), a protein controlled by the oxidative stress regulator OxyR [8,9,10,11]. *Deinococcus radiodurans* has two Dps proteins (i.e., Dps1, Dps2) and can survive through DNA protection under a number of extreme stresses (e.g., lengthy desiccation, intensive radiation, harmful chemical environments) [3,12]. *Deinococcus geothermalis* has been studied less than the genus type strain *D. radiodurans* [13], but a putative Dps gene *dgeo*_0257 has been identified; it should be noted this protein has not yet characterized nor matched to *D. radiodurans* Dps proteins. Importantly, *D. radiodurans* has one orthologous and hypothetical gene DR0582 with 72% identity. To characterize the functions of a Dps candidate protein of *D. geothermalis* (Dgeo_0257), a mutant strain with a disrupted target gene (Δ*dgeo*_0257) was constructed and tested for sensitivity to oxidative stress. During oxidative stress response experiments, a non-pigmented colony was observed in the *dgeo*_0257-disrupted background; we named this mutant strain ∆*dgeo*_0257 white mutant (aka ∆*dgeo*_0257w) because a wild-type strain generally is reddish in color. Since the *D. geothermalis* strain contains carotenoid—a carotene known to play a role in the protection of oxidative stress—it appears likely that this mutant strain has disruptions in the carotenoid-biosynthesis pathway. To support this hypothesis, we report evidence that a key enzyme of carotenoid biosynthesis was disrupted due to transposition of a unique type of transposase. For this reason, we have examined the possibility that DNA-protecting protein Dps controls the transposition of insertion sequence (IS) elements in genomic plasticity.

IS elements are frequently identified by their unique sequence features (e.g., transposase-encoding genes, terminal inverted repeats (TIR), and direct repeats (DR)) in the target sequence generated upon insertion [14,15]. These ISs are usually 750~2000 bp and encode enzymes causing transposition. *D. geothermalis* DSM11300^T^ has 93 transposases with 33 chromosomal, 38 within the plasmid pDGEO01, and 22 in the plasmid pDGEO02. Meanwhile, species within phylum *Thermus-Deinococcus*, including *D. radiodurans*, *D. deserti*, and *Thermus thermophiles* HB8 genomes, have 52, 17, and 92 ISs, respectively. In archaea, a halophilic archaeaon *Halobacterium salinarum* NRC-1 has 72 ISs. Amazingly, a crenarchaeon *Saccharolobus solfataricus* P2 genome has 443 ISs. All data were analyzed using ISfinder (www-is.biotoul.fr). In a recent report, *Thermus* mobilome for ISs distribution in *Thermus* spp. was characterized as active transposition of ISs induced by irradiation [16] like the *D. radiodurans* genome [17].

Here, we identified, for the first time in *D. geothermalis*, that the non-pigmented phenotypical transition due to the key enzyme of carotene biosynthesis *dgeo*_0524 (*crt*I) encoding a phytoene desaturase was inactivated by the transposition of an active IS element under oxidative stress conditions.

## 2. Materials and Methods

### 2.1. Bacterial Strains, Culture Conditions, and Construction of the Mutant

The strain *D. geothermalis* DSM 11300^T^ was obtained from the Korean Agricultural strains Collection Center (KACC12208). TGY medium containing 1% tryptone, 0.5% yeast extract, and 0.1% glucose was used for culturing *D. geothermalis* at 48 °C. *Escherichia coli* DH5α was used for transformation of recombinant DNA and grown on Luria-Bertani medium at 37 °C.

In order to disrupt a putative *dps* gene *dgeo*_0257, a mutant strain with a kanamycin-resistance cassette which was integrated into the target gene was constructed by homologous recombination. For the *dgeo*_0257 mutant strain, the homologous DNA sequence downstream of *dgeo*_0257 (roughly 1.0 kb) was amplified from genomic DNA using target-region primers and purified using a PCR purification kit (Bioneer, Korea). The purified DNA fragments and plasmid pKatAPH3 [18] were cleaved by *Xba*I-*Pst*I and ligated into a plasmid (named pKR0257). The homologous DNA sequence upstream of *dgeo*_0257 (roughly 1.0 kb) was amplified from genomic DNA using target-region primers and purified using a PCR purification kit (Bioneer, Korea). To yield pKRL0257, a vector which contained upstream and downstream regions of the *dgeo*_0257 gene, the purified DNA fragments and plasmid pKR0257 were digested with *Kpn*I-*Sal*I, ligated, and propagated in *E. coli*. Recombinant DNA (ie, pKRL0257) was purified from *E. coli* and transformed into *D. geothermalis* using a CaCl_2_-dependent technique described previously [19]. Wild-type *D. geothermalis* cultures were harvested in the exponential growth phase and suspended in TGY broth of 100 mM CaCl_2_: 100% glycerol (20:8:3, *v*/*v*/*v*). For transformation, 100 μL of the cell suspension and 5 μL of a solution containing pKRL0257 with ca 200 ng/ μL were added. The mixture was incubated on ice for 30 min and then at 32 °C for 30 min with gentle shaking. TGY broth (0.9 mL) was added into the mixture and incubated at 37 °C for 16 h with gentle shaking. Next, 100 μL of the mixture was spread onto TGY agar containing 8 μL/mL kanamycin for selection. The resulting transformed clone was identified by PCR analysis of the target gene region and named Δ*dgeo*_0257 mutant strain. Genome sequence of *D. geothermalis* DSM11300^T^ was used to design the following specific primers: chromosome (GenBank accession number NC_008025.1), plasmid 1 (NC_008010.2), and plasmid 2 (NC_009939.1).

### 2.2. Viability Test Studies in D. Geothermalis

The wild-type and Δ*dgeo*_0257 mutant strains of *D. geothermalis* were grown to an OD_600_ 1.0 in TGY broth at 48 °C. Similar number of cells from each culture were treated with 80, 100, and 120 mM hydrogen peroxide and incubated for 1 h. The stressed cells were serially diluted 10-fold in buffered saline from 10^0^ to 10^−5^. A 5-µL volume from each diluted suspension was spotted on the TGY agar plates and incubated at 48 °C.

### 2.3. PCR Detection of Transposition and Sequence Analysis

To detect transposition of ISs, we designed target-gene-encompassing primer sets with similar melting temperatures (Supplementary Table S1). Transposase-integrated sites were noted based on their size following PCR and agarose gel electrophoresis (i.e., enlarged in the non-pigmented mutant compared with wild-type and Δ*dgeo*_0257 mutant strains). The enlarged PCR products were then sequenced. The type of transposase, integrated sites, and specialized sequences including direct repeat and inverted repeat were determined. Transposition mode was also determined using PCR detection of genome distributed identical IS type genes with primer sets of outside border of the IS element.

### 2.4. RNA-Seq Analysis

To extract total RNA for RNA-Seq analysis, wild-type and the Δ*dgeo*_0257 mutant *D. geothermalis* strains were harvested at OD_600_ 4.0 in TGY broth at 48 °C. We manually used RIDOEx reagent (GeneAll, Korea) for extraction of total RNA. The extracted total RNA was purified using an RNeasy Mini Purification Kit and RNase-Free DNase I Set (Qiagen, Hilden, Germany). We commissioned *D. geothermalis* bacterial RNA-Seq and results analysis in e-biogen Co (Seoul, Korea). Total RNA quality measured by Agilent’s 2100 Bioanalyzer system and sequenced by Illumina HiSeq 2500 platform. Total raw read counts of wild-type and Δ*dgeo*_0257 mutant strains were 20,705,663 and 19,722,792, except plasmid pDGEO02, respectively. RNA-Seq analysis was performed using ExDEGA analysis tool of e-biogen Co.

### 2.5. Quantitative Reverse Transcriptase (qRT) PCR

To determine the expression levels of target transposase genes and related genes, we performed qRT-PCR as previously reported [20]. After cells were harvested at OD 4.0: (1) total RNA was extracted using a phenol-based RNA extraction procedure, (2) cDNAs were synthesized by PCR using reverse transcriptase, and (3) the synthesized cDNA were quantified and stored at –70 °C until real time PCR. Quantitative PCR was conducted using the Bio-Rad RT-PCR model CFX96TM Optics Module. The data were calculated using the qRT-PCR formula.

## 3. Results

### 3.1. Construction of the D. Geothermalis Δdgeo_0257 Mutant Strain

**Figure 1:**
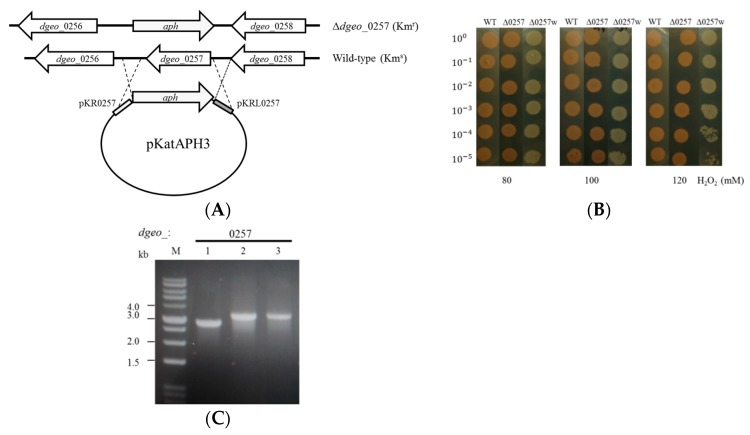
Scheme of construction of the Δ*dgeo_*0257-disrupted mutant strain (**A**) and viability assay of the wild-type, Δ*dgeo*_0257, and Δ*dgeo*_0257w mutant strains when treated with H_2_O_2_ (**B**). (**C**) PCR detection was done by encompassing primer sets for *dgeo*_0257. Lanes: 1, wild-type; 2, Δ*dgeo*_0257 mutant; 3, Δ*dgeo*_0257w mutant.

To identify physiological roles (e.g., oxidative stress resistance, DNA protection) of Dgeo_0257, a putative Dps protein, we built a recombinant plasmid carrying the *dgeo*_0257 gene-disrupted construct in the cloning vector pKatAPH3 (Figure 1A). This construct was generated by replacing the *dgeo*_0257 locus with the kanamycin-resistant *aph* gene via homologous recombination of a roughly 1 kb segment in both border regions of the target gene. The selected mutant clones with kanamycin resistance were confirmed by PCR (based on an increase in amplicon size). When growth patterns were compared between wild-type and mutant strain in TGY medium at optimum temperature, both strains revealed identical growth curves (data not shown), confirming that *dgeo*_0257 is not essential for cell growth.

### 3.2. The Effect of H_2_O_2_ on the Viability of the Δdgeo_0257 Mutant Strain

To characterize any potential differences between the viability of wild-type and Δ*dgeo*_0257 mutant strains, cells were grown under oxidative stress conditions (i.e., treatment with H_2_O_2_). After H_2_O_2_ treatment (80, 100, 120 mM final concentration) for 1 h, the viability of the wild-type strain was similar to the Δ*dgeo*_0257 mutant strain at all concentrations (Figure 1B). Therefore, we conclude that *dgeo*_0257 gene products are not directly involved in cell viability by a DNA protection procedure under oxidative stress conditions.

The results of the oxidative stress assay of the Δ*dgeo*_0257 mutant and wild-type strains revealed a non-pigmented colony on diluted spread plate after H_2_O_2_ treatment. The white colony, which we named Δ*dgeo*_0257w, grew well grown at 48 °C. Using 16S rRNA sequence analysis, we confirmed that the sequence of the 16S rRNA subunit is identical to *D. geothermalis* (i.e., 100% homology to the 16S rRNA sequence of the Δ*dgeo*_0257 mutant). This datum suggests that the non-pigmented strain Δ*dgeo*_0257w originated from Δ*dgeo*_0257 mutant but not from the wild-type (Figure 1C). Interestingly, the non-pigmented strain revealed slight sensitivity on oxidative stress (Figure 1B).

### 3.3. Detection of Transposition in Non-Pigmented Strain

**Figure 2:**
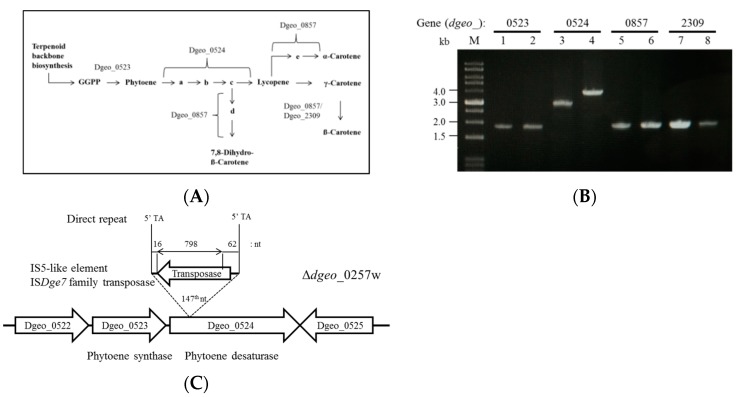
Scheme of carotenoid biosynthesis pathway and detection of transposition locus. (**A**) A simplified carotenoid biosynthetic pathway is illustrated, and genes encoding metabolic enzymes are indicated. Symbolized compounds: GGPP, geranylgeranyl pyrophosphate; a, Phytofluene; b, ζ-Carotene; c, Neurosporene; d, β-Zeacarotene; e, δ-Carotene. (**B**) PCR detection of transposition loci on four target genes for carotenoid biosynthesis. Lanes: 1, 3, 5, and 7 samples and 2, 4, 6, and 8 samples were amplified from Δ*dgeo_*0257 and Δ*dgeo_*0257w genomic DNA, respectively. (**C**) IS integration sites were determined by sequencing of *dgeo*_0524 gene amplicons.

In general, *Deinococcus* strains are orange in color resulting from carotenoid production, a pigment which is also involved in the oxidative stress response. The non-pigmented strain is likely the result of dysfunction of a key carotenoid pathway enzyme [21] (Figure 2A). We examined the corresponding pathway at KEGG database (https://www.genome.jp/kegg/) and chose the four genes (*dgeo*_0523, 0524, 0857, and 2309) to search for possible transposition among these genes by PCR with target gene–encompassing primers. Interestingly, *dgeo*_0524 gene (i.e., phytoene desaturase) was disrupted by integration of an IS element (Figure 2B). Thus, as hypothesized, disruption of a key enzyme in the carotenoid synthetic pathway created the observed non-pigmented phenotype.

Inserted DNA sequence analysis revealed the exact insertion site and facilitated the characterization of the inserted DNA sequence (Figure 2C). The insertion sequence is an IS*Dge*7 family IS element which has 5′-end 64 nt extension and 3′-end 16 nt extension with 798 nt ORF, 265 amino acids, of IS5 type transposase. This IS*Dge*7 type IS element has an 8 nt TIR sequence of ‘GAGGCTGG’ on both ends of the boarder. This sequence was integrated at the 147th nucleotide of *dgeo*_0524 on counter transcriptional direction with 2 nt DR sequence of “TA.” The *D. geothermalis* genome contains four identical IS*Dge*7 chromosomal family transposases, Dgeo_1042, 1699, 2208, and 2276 (Supplementary Table S2). The transposition pattern was determined by target-gene amplification using gene-specific primers at outside boarder of IS elements (Supplementary Table S1). All four target genes were still located at their original sites (Supplementary Figure S1A). Therefore, transposition of IS*Dge*7 family IS element occurred via replicative mode.

### 3.4. RNA-Seq Analysis of Δdgeo_0257 and Δdgeo_0257w and Identification of New Transpositions

To characterize the gene expression levels and patterns of Δ*dgeo*_0257 and Δ*dgeo*_0257w mutants, RNA-Seq analysis was performed on both mutant and wild-type strains. Interestingly, 10 IS elements of family IS*Dge*5, located on the chromosome and plasmid pDGEO01, have identical nucleotide sequences contain a transposase that was up-regulated. The mean expression level was 7.48-fold except *dgeo*_1807 of 3.49-fold (Supplementary Table S2).

**Figure 3:**
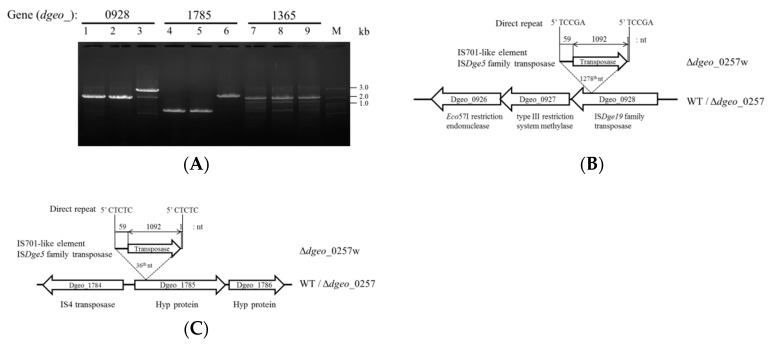
Detection of integrated ISs on substantially down-regulated candidate genes. (**A**) PCR amplification on three candidate genes (ie, *dgeo*_0928, 1785, 1365). Transposition by integrated ISs was detected in *dgeo*_0928 and *dgeo*_1785 regions. Lanes: 1, 4, and 7 were wild-type strain; 2, 5, and 8 were Δ*dgeo_*0257; 3, 6, and 9 were Δ*dgeo_*0257w genomic DNA. B-C, An IS*Dge5* family member IS element was integrated into both *dgeo*_0928 (**B**) and *dgeo*_1785 (**C**) genes.

In the non-pigmented colonies, however, the expression levels of four transposases belonging to IS elements of the IS*Dge*7 family were not affected (i.e., expression level of roughly 1.0). Thus, we aimed to identify transposition of up-regulated IS*Dge*5 family members of the IS701 type using strictly down-regulated genes with less than 0.3-fold between Δ*dgeo*_0257 and Δ*dgeo*_0257w mutants and selected four genes, *dgeo*_0926, 0927, 1785, and 1365 regions (Table 1). An extended near region *dgeo*_0928 with 6.20-fold was shown. Using a target-gene amplification assay, we identified two additional transposition target sites on *dgeo*_0928 and *dgeo*_1785 region but not on dgeo_0926, 0927, and 1365 as down-regulated targets (Figure 3A & Table 1). Surprisingly, both transpositions involved elements of the IS*Dge*5 family. The IS element integrated at 1278th and 36th nucleotide of the *dgeo*_0928 and *dgeo*_1785 genes, respectively (Figure 3B,C). The IS element of IS*Dge*5 family has 5′-end 59 nt extension and 3′-end 1 nt extension encompassing a 1092 nt, 363 amino acids long, IS701-like transposase. Unusually, both ends of border have 16 nt TIR sequence of ‘CTCAGGAGTTGCACCT’. The 3′ end of the TIR sequence was involved in transposition within the ORF region containing a stop codon. We also detected a transposition pattern by target-gene amplification using region specific primers. Eight target genes were still located at their own sites except for *dgeo*_2108 and *dgeo*_2659 (Supplementary Figure S1B). These two genes were not amplified in any of the three strains. It is possible that these genes were wrongly annotated or lost within the tested strains. Thus, the need to further define target genes from original stock collections and compare gene loci among them remains. From border sequence analysis of IS insertion, the duplicated DR sequences were 5 nt in length with completely different sequences (Supplementary Table S2). DR sequences of IS*Dge*5 family transposase in *dgeo*_0928 and *dgeo*_1785 integration were “TCCGA” and “CTCTC,” respectively. Therefore, the up-regulated IS*Dge*5 family transposases were also moved by replicative transposition with nonspecific integration within the background of a putative Dps gene *dgeo*_0257 mutant.

### 3.5. Detection of Expression Levels of Transposases on Oxidative Stress Condition

**Figure 4:**
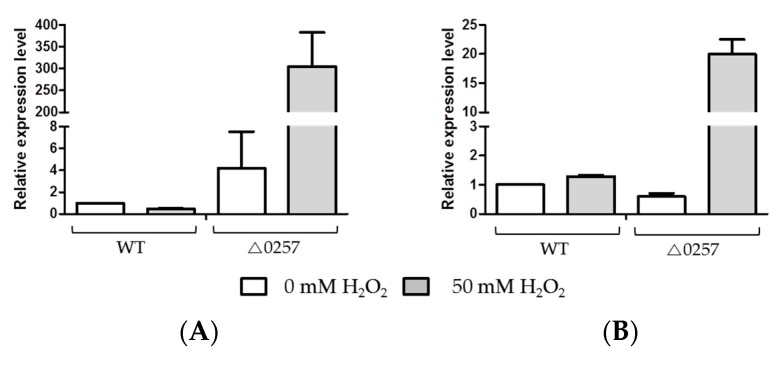
Comparison of relative expression levels of IS*Dge5* & IS*Dge7* family transposases upon treatment with/without H_2_O_2_ by qRT-PCR. Relative expression levels of IS*Dge5* family member transposases (**A**) and the IS*Dge7* family member transposase (**B**) in wild-type and Δ*dgeo_*0257 strains in the presence or absence of 50 mM H_2_O_2_. The bars indicate relative expression levels compared to those of the wild-type-strain in the absence of H_2_O_2_.

To characterize the expression levels of IS*Dge*5 family and IS*Dge*7 family transposases under oxidative stress conditions (i.e., treatment with 50 mM H_2_O_2_), quantitative RT-PCR was conducted for selected candidate IS members of IS*Dge*7 and IS*Dge*5 family in wild-type and Δ*dgeo*_0257 mutant strains. Relative expression levels of IS*Dge*7 and IS*Dge*5 family transposases were indicated 4.20 and 0.60-fold in Δ*dgeo*_0257 mutant strain under H_2_O_2_ absent condition. When treated with 50 mM H_2_O_2_, expression levels of IS*Dge*7 and IS*Dge*5 transposases indicated 0.49- and 1.29-fold in wild-type but strongly increased 304.99- and 19.96-fold in the Δ*dgeo*_0257 mutant strain, respectively (Figure 4). Thus, the expression of both IS*Dge*5 and IS*Dge*7 family transposases substantially increased under oxidative stress conditions in the Δ*dgeo*_0257 strain. It is possible that the induction of transposable element insertion sequences depends on regulatory machineries via a certain DNA-binding protein (e.g., Dps). The up-regulated transposases were more integrated into other sites within the *dgeo*_0257-disrupted mutant strain genome compared with the wild-type strain. Therefore, the putative Dps protein Dgeo_0257 may protect against induction of ISs and integration into genomic DNA by replicative transposition.

## 4. Discussion and Conclusions

Insertion sequences (ISs) are the smallest and most ubiquitous transposable element of mobile genetic elements within bacterial genomes [13,22]. ISs can be categorized into a family by combining several properties or their characteristics and have been recently classified [23,24]. Briefly, there are four major classification criteria: (1) the length and sequence of the perfect TIRs carried by many ISs at their ends, (2) the length and sequence of the short flanking DRs often generated within target insertion sites, (3) the organization of their open reading frame of transposases (e.g., DDE, DEDD, HuH motifs, tyrosine or serine specific recombinase relation), or (4) the target sequences into which they insert [24,25,26]. Nevertheless, there are miss-matched classifications of ISs type between GenBank and specialized program for analyzing ISs [eg, ISfinder (http://www-is.biotoul.fr)] [15]. For this analysis, we followed IS classification using the ISfinder program for ISs of *D. geothermalis*.

ISs act directly on the genome by moving within a specific genome or from cell to cell through gene transfer machinery. Therefore, ISs affect gene expression, genome shaping, and genome evolution [27]. Organisms with high transposition activity could often lead to severe down-regulation or disorder of gene expression. Because transposition of mobile genetic elements, including ISs and transposons, may cause serious harm to the host, there is a mechanism to prevent it. Inactive elements could also produce inactive transposases that can delay the transposition induced by active elements. DNA methylation or RNA interference are additional methods to reduce transposition activity [28]. The IS families within *E. coli* were analyzed by the 50-mutation accumulation (MA) method through generations without stressing *E. coli*. As generation progressed, no further transposition was caused by auto-regulation [29].

In general, the preferential target of IS insertion is in plasmids. Indeed, IS density is significantly higher in bacterial plasmids than in their host chromosomes [16,23]. In the *D. geothermalis*, IS density of plasmid was condensed with 66 copies/Mbp compared to 13 copies/Mbp of chromosome.

There are several reports summarizing IS transposition in *D. radiodurans*. The first identified IS2621 element of *Deinococcus* was found within the *uvr*A gene [30]. Later, IS8301 was detected in the *ppr*I gene [31], and in mutagenesis via IS transposition, multiple ISs were integrated within the *thy*A gene [32]. One of them, IS*Dra*2 is a member of IS200/IS605 family transposase and IS*Dra*2 transition is triggered approximately 100-fold by γ-irradiation [17,32].

Dgeo_0257 is a putative Dps protein and annotated as a ferritin-like protein, and in this study, we first characterized the transposition of IS*Dge*5 and IS*Dge*7 family transposases in the Δ*dgeo*_0257-disrupted mutant in *D. geothermalis*. The Δ*dgeo*_0257-disrupted mutant has been constructed to evaluate the physiological roles of this protein. When Δ*dgeo*_0257 mutant strain was treated with H_2_O_2_, its viability was not affected in the same way as the wild-type strain. After one of H_2_O_2_ treatments on the Δ*dgeo*_0257 mutant strain, we detected a non-pigmented colony. The white clone presented higher sensitivity under oxidative stress conditions. Consequentially, transposition of IS*Dge*7 occurred on phytoene desaturase, *dgeo*_0524, a key enzyme of carotenoid biosynthesis. Phytoene desaturase is an enzyme that converts the 40-carbon phytoene to lycopene. Nevertheless, these IS*Dge*7 type elements were not induced in the absence of H_2_O_2_ according to the RNA-Seq analysis data sets. Interestingly, IS*Dge*5 family transposases were upregulated in the same data sets. Thus, we tried to identify new transposition events on strictly down regulated genes. IS*Dge*5 transposase integrated into both *dgeo*_0928 and *dgeo*_1785 genes by replicative transposition with sequence non-specificity. At the moment, we are unable to explain why both *dgeo*_0926 and 0927 genes are substantially down-regulated in the Δ*dgeo*_0257w mutant strain (Table 1). Our results indicate that active transposition of IS*Dge*5 and IS*Dge*7 family IS elements occurred by oxidative stress (ie, H_2_O_2_ treatment) in the absence of the putative Dps protein Dgeo_0257 which resulted in disruption of functional genes. These transpositions are replicative action on genomic DNA. The Dgeo_0257 protein acts as a DNA protector against transposition of ISs. In the absence of the DNA-protection protein, a reddish *D. geothermalis* strain became non-pigmented by transposition of an IS element into a gene encoding phytoene desaturase, a key enzyme of carotenoid biosynthesis. In the future, the procedures used in this work will be further applicable to the study of transposition by other stress conditions (e.g., high γ-irradiation, temperature variations [33]) and other DNA damage agents). We also plan to determine the transposition rate of ISs in accumulated generations under oxidative stress conditions [29]. Those experiments may lead to a better understanding of the physiological pros and cons of transposition phenomena. Furthermore, we will investigate more exciting challenges, including whether the transposition induction of unique ISs was controlled in a special DNA-binding-protein-dependent manner.

## Figures and Tables

**Table 1:** List of down-regulated genes under 0.3-fold on expression ratio of Δ*dgeo_*0257w/Δ*dgeo_*0257 in RNA-Seq analysis.

Gene	Δ0257/WT	Δ0257w/WT	Δ0257w/Δ0257
*dgeo*_0927	2.35	0.21	0.09
*dgeo*_0926	2.10	0.28	0.13
*dgeo*_1785	0.61	0.09	0.15
*dgeo*_1365	1.58	0.45	0.28

## Supplementary Materials

Supplementary materials can be found at https://www.mdpi.com/2076-2607/7/10/446/s1.

## References

[1] Cox M.M., Battista J.R. (2005). *Deinococcus radiodurans*—The consummate survivor. Nature Rev. Microbiol..

[2] Slade D., Radman M. (2011). Oxidative stress resistance in *Deinococcus radiodurans*. Microbiol. Mol. Biol. Rev..

[3] Santos S.P., Mitchell E.P., Franquelim H.G., Castanho M.A.R.B., Abreu I.A., Romao C.V. (2015). Dps from *Deinococcus radiodurans*: Oligomeric forms of Dps1 with distinct cellular functions and Dps2 involved in metal storage. FEBS J..

[4] Battista J.R., Earl A.M., Park M.J. (1999). Why is *Deinococcus radiodurans* so resistant to ionizing radiation?. Trends Microbiol..

[5] Agapov A.A., Kulbachinskiy A.V. (2015). Mechanisms of stress resistance and gene regulation in the radioresistant bacterium *Deinococcus radiodurans*. Biochem. (Mosc)..

[6] Makarova K.S., Omelchenko M.V., Gaidamakova E.K., Matrosova V.Y., Vasilenko A., Zhai M., Lapidus A., Copeland A., Kim E., Land M. (2007). Deinococcus geothermalis: The pool of extreme radiation resistance genes shrinks. PLoS ONE.

[7] Lim S., Jung J., Blanchard L., de Groot A. (2019). Conservation and diversity of radiation and oxidative stress resistance mechanisms in *Deinococcus* species. FEMS Microbiol. Rev..

[8] Almiron M., Link A.J., Furlong D., Kolter R. (1992). A novel DNA binding protein with regulatory and protective roles in starved *Escherichia coli*. Genes Dev..

[9] Altuvia S., Almiron M., Huisman G., Kolter R., Storz G. (1994). The *dps* promoter is activated by OxyR during growth and by IHF and in stationary phase. Mol. Microbiol..

[10] Ceci P., Cellai S., Falvo E., Rivetti C., Rossi G.L., Chiancone E. (2004). DNA condensation and self-aggregation of *Escherichia coli* Dps are coupled phenomena related to the properties of the N-terminus. Nucleic Acid Res..

[11] Calhoun L.N., Kwon Y.M. (2011). Structure, function and regulation of the DNA-binding protein Dps and its role in acid and oxidative stress resistance in *Escherichia coli*: A review. J. Appl. Microbiol..

[12] Nguyen K.H., Smith L.T., Xiao L., Bhattacharyya G., Grove A. (2012). On the stoichiometry of *Deinococcus radiodurans* Dps-1 binding to duplex DNA. Proteins.

[13] Ferreira A.C., Nobre M.F., Rainey F.A., Silva M.T., Wait R., Burghardt J., Chung A.P., da Costa M.S. (1997). *Deinococcus geothermalis* sp. nov. and *Deinococcus murrayi* sp. nov., two extremely radiation-resistant and slightly thermophilic species from hot springs. Int. J. Syst. Bacteriol..

[14] Mahillon J., Chandler M. (1998). Insertion sequences. Microbiol. Mol. Biol. Rev..

[15] Siguier P., Perochon J., Lestrade L., Mahillon J., Chandler M. (2006). ISfinder: The reference centre for bacterial insertion sequences. Nucleic Acids Res..

[16] Blesa A., Sanchez M., Sacristan-Horcajada E., Fuente S.G., Peiro R., Berenguer J. (2019). Into the *Thermus* mobilome: presence, diversity and recent activities of insertion sequences across *Thermus* spp.. Microorganisms.

[17] Pasternak C., Ton-Hoang B., Coste G., Bailone A., Chandler M., Sommer S. (2010). Irradiation-induced *Deinococcus radiodurans* genome fragmentation triggers transposition of a single resident insertion sequence. PLoS Genet..

[18] Ohba H., Satoh K., Yanagisawa T., Narumi I. (2005). The radiation responsive promoter of the *Deinococcus radiodurans ppr*A gene. Gene.

[19] Brim H., Venkateswaran A., Kostandarithes H.M., Fredrickson J.K., Daly M.J. (2003). Engineering *Deinococcus geothermalis* for bioremediation of high-temperature radioactive waste environments. Appl. Environ. Microbiol..

[20] Kim M., Jeong S., Lim S., Sim J., Rhie H.G., Lee S.J. (2017). Oxidative stress response of *Deinococcus geothermalis* via a cystine importer. J. Microbiol..

[21] Tian B., Hua Y. (2010). Carotenoid biosynthesis in extremeophilic *Deinococcus-Thermus* bacteria. Trends Microbiol..

[22] Sawyer S.A., Dykhuizen D.E., DuBose R.F., Green L., Mutangadura-Mhlanga T., Wolczyk D.F., Hartl D.L. (1987). Distribution and abundance of insertion sequences among natural isolates of *Escherichia coli*. Genetics.

[23] Siguier P., Gourbeyre E., Chandler M. (2014). Bacterial insertion sequences: Their genomic impact and diversity. FEMS Rev..

[24] Siguier P., Gourbeyre E., Varani A., Ton-Hoang B., Chandler M. (2015). Everyman’s guide to bacterial insertion sequences. Microbiol. Spectrum..

[25] Palmenaer D.D., Siguier P., Mahillon J. (2008). IS4 family goes genomic. BMC Evol. Biol..

[26] Guerillot R., Siguier P., Gourbeyre E., Chandler M., Glaser P. (2014). The diversity of prokaryotic DDE transposases of the mutator superfamily, insertion specificity, and association with conjugation machineries. Genome Biol. Evol..

[27] Vandecraen J., Chandler M., Aertsen A., Van Houdt R. (2017). The impact of insertion sequences on bacterial genome plasticity and adaptability. Crit. Rev. Microbiol..

[28] Muñoz-López M., García-Pérez J.L. (2010). DNA transposons: nature and applications in genomics. Curr. Genom..

[29] Sousa A., Bourgard C., Wahl L.M., Gordo I. (2018). Rates of transposition in *Escherichia coli*. Biol. Lett..

[30] Narumi I., Cherdchu K., Kitayama S., Watanabe H. (1997). The *Deinococcus radiodurans uvr*A gene: identification of mutation sites in two mitomycin-sensitive strains and the first discovery of insertion sequence element from deinobacteria. Gene.

[31] Hua Y., Narumi I., Gao G., Tian B., Satoh K., Kitayama S., Shen S. (2003). PprI: A general switch responsible for extreme radioresistance of *Deinococcus radiodurans*. Biochem. Biophys. Res. Commu..

[32] Mennecier S., Servant P., Coste G., Bailone A., Sommer S. (2006). Mutagenesis via IS transposition in *Deinococcus radiodurans*. Mol. Microbiol..

[33] Ohtsubo Y., Genka H., Komatsu H., Nagata Y., Tsuda M. (2005). High-temperature-induced transposition of insertion elements in *Burkholderia multivorans* ATCC17616. Appl. Environ. Microbiol..

