# Supplementary material for: Transposition of Insertion Sequences was Triggered by Oxidative Stress in Radiation-Resistant Bacterium Deinococcus geothermalis
Source: Microorganisms. 2019 Oct 12;7(10):446. doi: 10.3390/microorganisms7100446 (PMC6843628; doi:10.3390/microorganisms7100446)
Supplement: Supplementary file 1 [file microorganisms-07-00446-s001.pdf]

Supplementary data

Transposition of insertion sequences was triggered by oxidative stress in radiation-resistant bacterium *Deinococcus geothermalis*

Chanjae Lee[§], Nakjun Choi[§], Min K. Bae, Kyungsil Choo and Sung-Jae Lee*

Department of Biology, Kyung Hee University, Seoul, 02447, Korea

Supplementary Table S1. List of primers used in this work

Primer	Sequence (5′→ 3′)
Construction of Dgeo_0257 mutants	
0257_LF_*Kpn*I	AAGGTACCTATCGCCATAAGCTCTAA
0257_LR_*Sal*I	AAGTCGACGCTCCAATTGTGGATTAA
0257_RF_*Xba*lI	AATCTAGAAAGAACTCCTTGACGTCGT
0257_RR_*Pst*I	AACTGCAGGGTTTTGGAAACTGCTTTA
Disruption of carotenoid gene	
0523_F	CTTGGCCTTGATGACGTTCG
0523_R	GAGAAAGAGAGCGGTGCAGAA
0524_F	CGTTGATGCGTGATCTCTCTG
0524_R	CATGTCAGGCAGTTTGCTCTAG
0857_F	GATGACCCGGTTGAGTGCAT
0857_R	CAGCGTGCGCTTTGAGAAATG
2309_F	GTCGCCGCCTTATATCCCAA
2309_R	CAGATGTTCGCTTTTCGCCC
Detection of IS*Dge*7	
1042_F	GTCGAGCATCACCACGTACT
1042_R	CTCGCTGGACCACTGATGAA
2208_F	GCACGTCTTTCGCGTATTCC
2208_R	GCCTGAATGGTCTGACTCTCC
1699_F	ACGGCACCCATACTTTCCTG
1699_R	GTAAGATGACGTCATGGACC
2276_F	GATGCTGACCGATTGACCGA
2276_R	TACGCCGAGCATGAGAACTC
Detection of IS*Dge*5	
0308_F	TGGTTCTGTGCCTGCGGAC
0308_R	ATTCAGCGTGCTGATGGTCCA
0464_F	CGGTGCGAACGGCCAGATTA
0464_R	AACGTGGAGCAGTGGAGATCA
0925_F	GCGCGACTATCCCGAACAAAT
0925_R	GAGGTGAAAGGCGGAGTTTGTTAT
1702_F	ATGGTTAAATGCCGAGCGGGA
1702_R	CATATCCAGACGACTCTGCGGT
1807_F	CACTCAGGTTGCGGCTGAG
1807_R	TAGAGCCTGTTTGTGACCTGCC
2108_F	ACCGCTTCTTCGCCTACTAC
2108_R	GGCGATGTAGTCAGAGAGGAGAT
2205_F	CCAAGACAACGAGAACGCTGATG
2205_R	AGGGCAAAAGCAGAGCAGTC
2430_F	CGGCAAAGATGAGAGACTTGTG
2430_R	CGCTCAACCAACATTGAACTGAG
2659_F	GCCTACCGGAACCAGATTGTGA

2659_R	CTGGCCCACCCAACCAAAT
2823_F	ACTGTACGCTCCTCTTTCATGGA
2823_R	AGAACTGACTTTGACACCGAAGAT
Down regulated genes	
0926-27_F	ATTCAGATGTCCGCCCACAA
0926-27_R	GGTCGAAGACAGCGTGCTTA
0928_F	GCCACACGCTGTTTGGATTT
0928_R	GCAACAATTTCACCTGCACGC
1785_F	GGTCTCCTACATTGCACAGT
1785_R	GATACCTGCATCCACATCAG
1365_F	AGTCCTCGGGAATGTTGTCC
1365_R	TAGTAAGTTCGCGTTCGCCC
qRT-PCR for transposase	
GAPDH_F	CACCGTTCACTCCTACACGA
GAPDH_R	AGGTGGGGATGATATTGATCG
IS*Dge*7_F	TCCGCGTCTACAACGAAGTC
IS*Dge*7_R	TTTCCCCGGTAGACCCGATA
IS*Dge*5_F	CAGGTGTGCTTTGCAGGGTA
IS*Dge*5_R	CGTGCATGATCTGCGACTGT

Supplementary Table S2. Comparison of gene induction levels of IS elements of the IS*Dge7* and IS*Dge5* families in the Δ*dgeo*_0257 and Δ*dgeo*_0257w strains. The relevant genetic features of these IS types are also indicated.

Type of IS element	Transposase gene	Folds (from RNA-Seq)	Loci	Direct repeat	Inverted Repeat
IS5(IS1031)-like element IS*Dge*7 family (878 nt)	*dgeo*_1042	1.09	Chr	TA	8 nt (GAGGCTGG)
	*dgeo*_1699	1.08	Chr	TA	
	*dgeo*_2208	1.08	Chr	TA	
	*dgeo*_2276	1.10	Chr	TA	
IS701-like element IS*Dge*5 family (1157 nt)	*dgeo*_0308	7.17	Chr	CAAGA	16 nt (CTCAGGAGTTGCACCT)
	*dgeo*_0464	8.86	Chr	ACCTG	
	*dgeo*_0925	6.84	Chr	CTCTT	
	*dgeo*_1702	6.53	Chr	CTCTA	
	*dgeo*_1807	3.49	Chr	TCAAG	
	*dgeo*_2108	8.46	Chr	GTATT	
	*dgeo*_2205	8.79	Chr	GCTGA	
	*dgeo*_2430	7.05	Plas1	TCGGT	
	*dgeo*_2659	6.85	Plas1	CATGA	
	*dgeo*_2823	6.73	Plas1	AGAAA	

Supplementary Figure 1

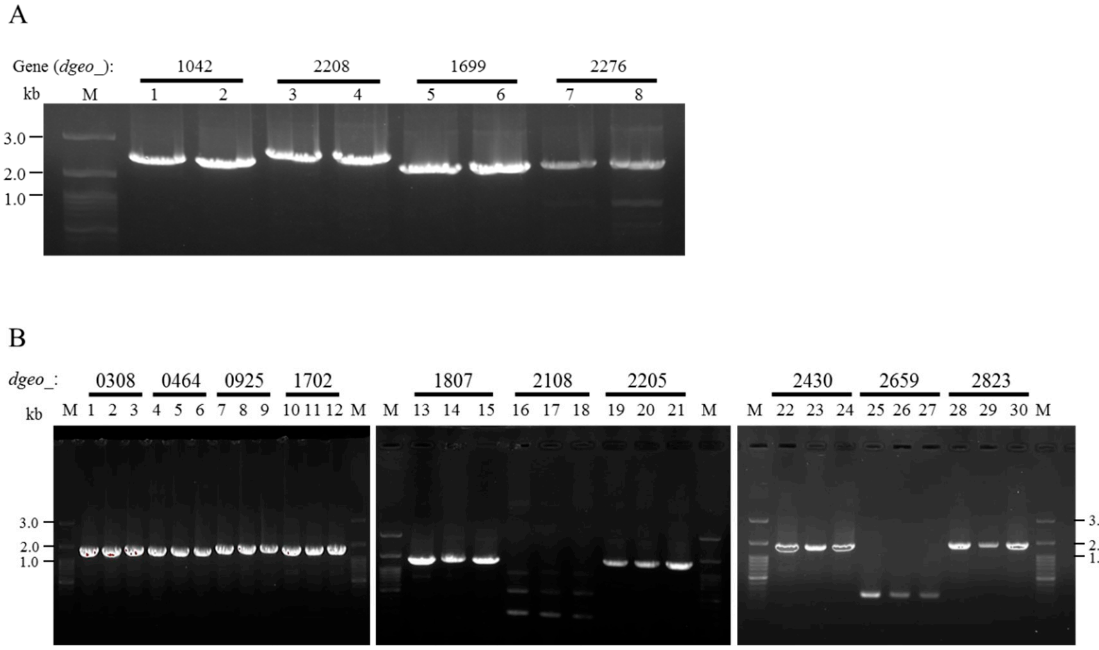

Figure S1. Detection of four IS*Dge7* (A) and ten IS*Dge*5 (B) family member transposases by encompassing primer sets in Table S1. A, Lanes: 1, 3, 5, and 7 samples and 2, 4, 6, and 8 samples were amplified from wild-type and Δ*dgeo*_0257w genomic DNAs, respectively. B, Lanes: 1, 4, 7, 10, 13, 16, 19, 22, 25, and 28 were amplified from wild-type genomic DNA; 2, 5, 8, 11, 14, 17, 20, 23, 26, and 29 were amplified from Δ*dgeo*_0257 genomic DNA; 3, 6, 9, 12, 15, 18, 21, 24, 27, and 30 were amplified from Δ*dgeo*_0257w genomic DNA. Both *dgeo*_2108 and *dgeo*_2659 genes were lost from present *D. geothermalis* genome. These data indicate that the action of transposition of both IS*Dge*7 and IS*Dge5* family member transposases is replicative mode.
